# The utility of delayed-enhancement and t2-weighted cardiovascular mri for predicting clinical outcomes in patients at high risk for cardiac sarcoidosis

**DOI:** 10.1186/1532-429X-13-S1-O105

**Published:** 2011-02-02

**Authors:** Yongkasem Vorasettakarnkij, Hector M Medina, Waleed Ahmed, Godtfred Holmvang, Peerawut Deeprasertkul, Daniel J Verdini, Shanmugam Uthamalingam, Thomas J Brady, Brian B Ghoshhajra, David E Sosnovik

**Affiliations:** 1 Martinos Center for Biomedical Imaging, Massachusetts General Hospital, Harvard Medical School, Charlestown, MA, USA; 2 Cardiac MRI/PET/CT program, Department of Radiology, Massachusetts General Hospital, Harvard Medical School, Boston, MA, USA; 3 Cardiology Division, Massachusetts General Hospital, Harvard Medical School, Boston, MA, USA

## Purpose

To evaluate the utility and prognostic value of delayed enhancement and T2 weighted cardiovascular MRI in patients at high risk for cardiac sarcoidosis.

## Introduction

The utility of delayed-enhancement (DE) has been extensively studied in patients with sarcoidosis who are asymptomatic or at low risk for cardiac involvement. However, there is lack of data regarding the utility of DE in patients with sarcoidosis who present with high grade cardiovascular symptoms. Also, the utility of T2-weighted imaging has not been evaluated in these patients.

## Methods

Patients referred for cardiac MRI (CMR) from 01/01/2003 to 12/31/2009 in our institution, and who had biopsy proven sarcoidosis without concomitant coronary artery disease, were included. Patients were classified as presenting with a major rhythmic disturbance (MRD) if the referral for cardiac MRI was prompted by high grade atrio-ventricular block, sustained ventricular tachycardia or ventricular fibrillation. Mean duration of follow up after MRI was 20.7 +13.8 months.

## Results

**Figure 1:**
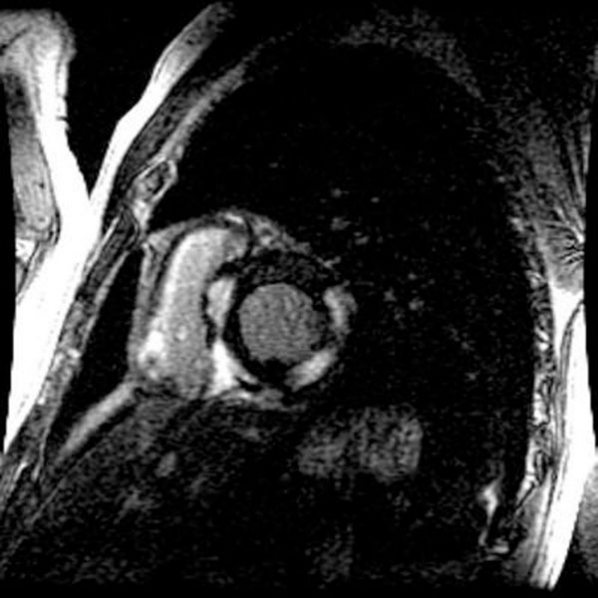
Delayed-enhancement of a patient presenting with complete heart block

Forty patients with sarcoidosis referred for CMR met entry criteria. 8/40 (20%) patients experienced a MRD prior to CMR. Of these, 8/8 (100%) patients demonstrated hyper-enhancement and 4/8 (50%) had hyper-intensity on T2W-CMR. During follow-up, 8/8 (100%) had recurrent MRD or required permanent pacemaker implantation. DE of a patient presenting with complete heart block is shown in Figure 1. In the 32/40 (80%) patients without an MRD prior to MRI, 11 (34%) demonstrated hyperenhancement on DE-MRI. From these, 3/11 (27%) also had hyper-intensity on T2W-CMR. In 21/40 (53%) patients presenting without a prior MRD and no delayed enhancement, T2W-CMR was negative in all cases. None of these 21 patients had a MRD during follow-up.

## Conclusions

In patients with suspected cardiac sarcoidosis who present with a MRD, DE-CMR is extremely likely to be positive and is helpful in confirming cardiac involvement. These patients are highly likely to experience recurrent adverse events regardless of the presence or absence of acute edema or inflammation on MRI. Patients without a prior MRD who show no evidence of delayed enhancement by CMR have an excellent prognosis and are at very low risk of developing a MRD within next year. A history of a prior MRD is the strongest predictor of a future MRD. CMR, however, has a valuable role to play in confirming the diagnosis and refining risk, particularly in those presenting without a prior MRD.

